# Electricity in Dentistry

**Published:** 1897-05

**Authors:** W. Herdman

**Affiliations:** University of Michigan


### ELECTRICITY IN DENTISTRY.

### Electricity in Dentistry.

BY W. HERDMAN, UNIVERSITY OF MICHIGAN.

Dentistry should be regarded, if it is not, as one of the specialties in the practice of medicine and surgery. The best Schools of Dentistry do so regard it and the curriculum of these schools provides for a fundamental training such as is required of the medical student. A thorough knowledge of chemistry, anatomy, physiology, hygiene, bacteriology, histology, pathology and materia medica is quite as necessary for the efficient practice of dentistry as of medicine and surgery. the field of operation for the dentist, though limited, is not any more so than that of the laryngologist, rhinologist, oculist or aurist. The variety of pathological conditions the dentist has to meet and contend with is quite as great as in any one of these other specialties and the resources he should have at his command, in order to successfully check and counteract disease in his peculiar field, are much the same. Especially is he in need of the help electricity furnishes, for not only can he get valuable aid from it as a motive power for various mechanical devices which are peculiar to dentistry and for which electric motors are best adapted, such as drills, burrs, burnishers, mallets, etc., but, as a source of light for exploring the mouth, and of heat, when this is wanted for strictly limited application, electric means are far superior to any other.

But little need be said in these columns calling the dentist's attention to the advantages to be gained from using electricity as a substitute for driving motors or for furnishing heat for cauteries or the hot air syringe or lighting exploring lamps, since the manufacturers of such appliances can be trusted to bring the merits of such devices to the attention of their customers and use will soon settle the question of superiority.

It is to the more strictly therapeutic applications of electricity to dentistry that we would direct the attention of the readers of the *Bulletin* in the present number. Many inquiries have, from time to time, been addressed to us upon this subject. The demand for such knowledge, we find, is great and the sources of information limited.

Among the various forms of electric energy that have been considered in more or less detail in former numbers of the *Bulletin*, i. e. the direct current or galvanism, the induced current or faradism, and static electricity, the former of these, the direct current, is the one to be chiefly if not solely considered, since it is the one most capable of effecting certain desirable changes in those tissues with which the dentist has to deal.

The induced current or farradism may be of some service in allaying pain or obtunding sensitive dentine or in stimulating nutritive activity in the pulp, gums or alveoli while the direct or galvanic current can not only do this, but, in addition, its range of electrolytic and cataphoric action gives to it a much wider field of usefulness with which the dentist should become familiar. We will endeavor in the following articles to point out some of the applications of the direct current to dentistry:

---

### ELECTROLYSIS IN DENTISTRY.

While treating of Electrolysis in No. 2, Vol. I, of the *Bulletin*, attention was called to the difference of effect upon living tissues produced at the anode or positive electrode, from that at the cathode or negative electrode when a constant or direct current of even a few milliamperes is passed for a few minutes.

The fluids of the tissues and the substances in solution in them are decomposed. Oxygen, the acids and bromine, chlorine, iodine, etc., accumulate in the vicinity of the anode, while hydrogen, the alkalies and metals gather at the cathode.

It is possible then for the dentist, as it is for the physician, to utilize this capacity of an electric current, and to so localize its action upon any point with such precision as to increase the acidity or alkalinity of the tissue at that point, according to the pole used.

By this method of modifying the distribution of the constituents of the tissues nutrition may be changed for better or for worse, and the electrolytic action may be carried to such a degree at either electrode as to bring about extensive destruction of tissue.

Sometimes it is such destruction of tissue that the dentist desires. When that tissue is an abnormal growth, having its origin from any one of the tissues of the jaw, as a myeloma, no simpler or more efficient means can be employed for its removal than that of electrolysis. A strong needle may be introduced into the base of such a growth at the line where the normal tissue verges upon the abnormal, and such needle be made the positive electrode of a direct or galvanic current, of a strength of five or eight milliamperes. This will, in a few moments of application, produce such change in the vicinity of the needle as to check the further development of the growth at that point, and if the needle is applied at several points about the margin of the growth during the one treatment, and the process, if necessary, be repeated a few times, the growth will undergo retrograde changes and disappear.

All kinds of abnormal growths that occur in the region of the mouth, no matter what their structure, are amenable to this disintegrating action of electrolysis, and by means of it their removal can be effected with the least possible loss of normal tissue, for with suitable appliances, and skill in handling them, the destructive electrolytic action can be limited with the utmost nicety to the abnormal tissue only.

For such abnormal growths as are soft and vascular in structure the styptic, coagulating action of the anode is best adapted, while for hyperostoses, fibrous or cartilaginous overgrowth cathodal electrolysis, which causes softening and liquefaction, would be more suitable and be followed by resolution more promptly.

The strength of current that will effect these electrolytic changes in tissue is not great. A current of three or five milliamperes, continued for five or eight minutes, where the active electrode is a needle point, will so far devitalize the tissue in the immediate vicinity of the needle as to result in gradual disintegration and absorption at that point. A stronger current will result in immediate destruction of tissue, which will give a more noticeable change at the time of operation, but the result may be an extensive slough with a resulting sore and subsequent eschar, which is oftentimes undesirable. So that feebler applications, more frequently made, if need be, effect more satisfactory results by instituting in the part a slow process of disintegration to which the normal tissue may adjust itself, and replace by normal growth rather than by scar tissue, as would be the case after extensive sloughing.

*Metallic Electrolysis:* The metal point or needle which is made use of for an electrode may itelf be readily acted upon at the anode by the oxygen and chlorine that is set free at that pole by the electrolytic process, and an oxy-chloride of the metal is formed which of itself has beneficial therapeutic action which may be made use of when the pathological condition is such as to require it. The oxy-chloride of copper, zinc or silver are all of them styptic and antiseptic in character, and this method of employing them, that is, by having the anode made of one or other of these metals, is both a convenient and efficient way for getting the medicinal effect of these salts upon any part. When employing electrolysis for any purpose, the direct current used should be completely under the control of the operator, and should be so managed as to give no disagreeable shock to the patient. This result can be readily attained if one makes use of reliable apparatus and is thoroughly informed as to how to use it.

Before the current is turned on the active electrode should be introduced into or brought in contact with the part to be electrolyzed, and the other, or dispensing electrode, which ought to be of large size and well moistened, should be in place, either upon the hand of the patient, the back of the neck, or over the breast-bone, and then the current should be by the gentlest graduation turned on by means of a rheostat or controller, until the milliamperemeter indicates the desired quantity, and when the application is ended the current should be as gradually withdrawn. Operating in this manner the patient experiences no shock or special discomfort, especially if the precaution is taken both by patient and operator during the application, to make no break in the circuit at either electrode.

---

### CATAPHORESIS AND ANAPHORESIS.

Cataphoresis is a term used to designate that action of a direct or galvanic current upon the tissues of the living organism by which substances placed upon the anode are, by the moving power of the current, driven toward the cathode, and so carried for a greater or less distance into the tissues. By this means medicinal substances which would not, by mere contact, find their way into the tissues, are made to do so, and their peculiar effects upon the tissue, either locally or over a wide range, as the entire surface of the body, may be brought about. Since the adoption of the term cataphoresis much has been learned concerning this action of the direct current, which shows that the first conceptions that were entertained concerning it are entirely too narrow. It was thought that this moving power of the current upon substances, in solutions which were made a part of the circuit, acted only in what is assumed to be the direction of the current, that is, from anode to cathode. It was supposed that a substance, in order to be conveyed through the tissues by means of the current, must be placed upon the anode. But later investigations have shown that this is true only of certain substances, and that others may be made to penetrate the tissues with equal facility if placed upon the cathode, the electric action driving them in a direction opposite to that in which the current is supposed to be traveling, from cathode to anode. The word *anaphoresis* has been suggested as an appropriate term by which to designate this share in the transmission of substances by means of the direct or galvanic current, and the appropriateness of the term recommends it for general adoption. Anaphoresis will therefore be henceforth used in the columns of this *Bulletin* to designate the transmission of medical substances from cathode to anode by means of electric currents.

*Relation of Cataphoresis and Anaphoresis to Electrolysis.*—Since it has been found that the nature of a substance determines the direction it will travel in a solution, when that solution is made a part of the path of a direct current, strong evidence has been educed to prove that what we term cataphoresis and anaphoresis is nothing more or less than the migration of the ions in the process of electrolysis; the positively charged ions seeking the negatively-charged ions being attracted to the positive pole.

Many experiments arranged to simplify and analyze the action of these migrations of various substances through the influence of direct currents seem to justify by their results the conclusion that electrolysis has much to do with it.

But in order to explain, by electrolysis, all that occurs in the moving of substances in the one or other direction by means of the electric current, the present conception of the electrolytic process must be enlarged so as to include not only the migration of ions which have become disassociated from chemical combinations through the action of the passing current, but also the migrations of molecules of complex composition that are suspended in the solutions, and which do not break up into ions prior to obeying the moving power of the current, but travel in their original form, some in one direction, some in another, according to their nature. Thus, certain of the coloring matters, such as methylene blue, are made to move from anode to cathode by the action of the current, when suspended in solution in the current's path, while eosin travels from cathode to anode. Many substances, also, which are, by currents of certain strength, broken up into ions and then obey the law of ionic migration, are found to act differently if the current is not of sufficient strength to cause electrolytic decomposition. The molecule of the substance, undecomposed, is found to move in one or other direction, according to its nature and composition, under the influence of these weaker currents. This we think we have, by experiment in this laboratory, proved to be true of potassium iodide and cocaine hydrochlorate, both of which are readily decomposed by the action of moderately strong currents, but the molecule of each may, to a certain extent, be transmitted intact by weaker currents.

Only by experiment can it be determined whether a substance is cataphoric or anaphoric. And, likewise, the strength and density of current necessary to cause a substance to penetrate the tissue as an unbroken molecule, or in the form of ions resulting from it by electric disassociation, must be settled by actual test. —*Bulletin.*

